# Troubleshooting the Poor Flow Problem of Valsartan Drug Powder Using the Crystallo-Co-Agglomeration Technique

**DOI:** 10.7759/cureus.38590

**Published:** 2023-05-05

**Authors:** Madan Mohan Gupta, Arindam Chatterjee, Tarachand Kumawat

**Affiliations:** 1 School of Pharmacy, Faculty of Medical Sciences, The University of the West Indies, St. Augustine, TTO; 2 Department of Pharmaceutics, Jaipur College of Pharmacy, Jaipur, IND; 3 Department of Pharmaceutics, Regional College of Pharmacy, Jaipur, IND

**Keywords:** crystallo-co-agglomeration, tablet, spherical crystal, valsartan, antihypertensive

## Abstract

Pharmaceutical tablets are a popular solid dosage form and have a significant ratio in the available solid dosage forms. They are a popular choice for patients due to the ease of administration as well as for pharmaceutical manufacturers due to the low cost of manufacturing, packaging, and other pharmaceutical parameters. However, the drug powder should either be crystalline or turned into a granular form using wet-dry granulation techniques to improve the flow and compressibility. The valsartan drug, which is commonly used as an antihypertensive drug, is an amorphous drug and has an angle of repose of more than 40º. Therefore, it needs to be converted into a granular form. This work uses the spherical crystals of the valsartan drug because they flow well and can be used for pharmaceutical tablets. Different process parameters, such as mixing speed, mixing time, and temperature, were optimized to obtain effective process parameters. The final batch of spherical crystals of valsartan had an angle of repose of 27.23º, which shows that prepared spherical crystals flow well.

## Introduction

Pharmaceutical tablets can be prepared using either the direct compression method or the granulation method using wet or dry granulation. To prepare a pharmaceutical tablet, two properties, that is, flowability and compressibility, are important, and these two properties are available in granular and crystalline powder, and because of this, it is required that drug powders should be crystalline or granular [1-3]. The process of converting amorphous powder into granular form using either wet or dry granulation requires many steps, and all these steps need to be optimized and validated. The validation process itself takes a lot of time, and because of this, the final product is expensive. Another method, which is economical, is to convert amorphous powder into spherical crystals using the crystallo-co-agglomeration technique to create spherical crystallization. This method combines crystallization, agglomeration, and spheronization in a single step and is a very effective technique to convert fine powder into spherical crystals that can be used in pharmaceutical tablets [4-9]. The valsartan drug, which is an antihypertensive and available in an amorphous form to prepare pharmaceutical tablets, can be prepared using spherical crystals. Valsartan drug is a white or almost white hygroscopic powder that is practically insoluble in water, freely soluble in anhydrous ethanol, and sparingly soluble in methylene chloride. This drug, according to the Biopharmaceutical Drug Classification System, is a class II drug and is characterized by low solubility and high permeability. Further, it mainly displays dissolution-dependent oral bioavailability [10-15].

## Materials and methods

Valsartan drugs and other chemicals are procured from local suppliers of the analytical category. The following instruments were used: a digital weighing balance (Shimadzu, Japan), an FTIR spectrometer (Shimadzu, Japan), a tap density tester USP (Veego, India), a UV-VIS spectrophotometer (Shimadzu, Japan), and a differential scanning calorimeter (Shimadzu, Japan).

Drug profile

**Figure 1:**
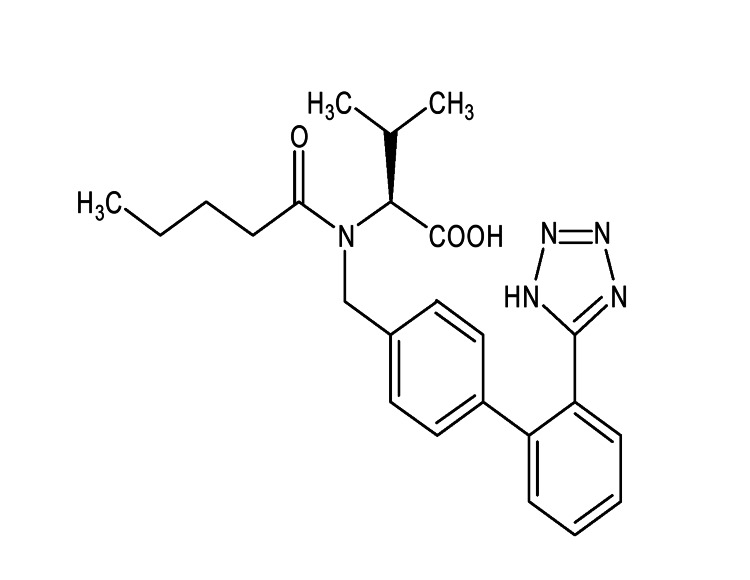
Valsartan drug

Valsartan drug, which is an antihypertensive drug, is shown in Figure 1.

This drug falls under the category of antihypertensive drug in the class of angiotensin antagonists. The International Union of Pure and Applied Chemistry name of this drug is 3-methyl-2-[pentanoyl-[4-[2-(2H-tetrazoyl-5-yl) phenyl]phenyl]methyl]amino]-butanoic acid, and its molecular formula is C24H29N5O3. The drug appears as a white crystalline powder with a melting point of 116-117°C. The drug is very soluble in methanol and practically insoluble in water. The pharmacokinetic parameters of the drug are T1/2 (oral) = 7.05 hr, Cmax (mg/ml) = 1.64, bioavailability = 23%, and 94%-97% protein bound.

Drug analysis and characterization of valsartan drug

For every formulation and analysis, the certificate of analysis must be checked to confirm the drug. This was also necessary to confirm the identity of the valsartan drug. For identification, two different analytical methods, that is, FTIR spectroscopy and differential scanning calorimetry (DSC), were used. The peaks and melting points were checked against the standard given values and found to be the same.

Flow property of valsartan

The valsartan drug is a poorly flowable drug. Therefore, to confirm the flow property, it was checked by the angle of repose method. The angle of repose of valsartan drug powder was 41.92º. The angle of repose was checked using the fixed funnel method. The fixed amount of valsartan drug was poured into the glass funnel, and the radium and height of the heap were checked to calculate the angle of repose. The standard range of the angle of repose is given in Table 1. The equation used was θ = tan-1 (h/r).

**Table 1:** Standard range of the angle of repose

Angle of repose	Type of flow
25-30	Excellent
31-35	Good
36-40	Fair
41-45	Passable
45	Poor

Preparation of the spherical crystals

To prepare good-quality spherical crystals, it is required to select the bridging liquid and the mode of addition of the bridging liquid and to optimize the agitation time and speed.

Selection of bridging liquid

Various bridging liquids are required to convert amorphous powder into crystalline powder: toluene, chloroform, and dichloromethane.

Mode of addition of bridging liquid

The effect of the mode of addition of the bridging liquid, which is a process parameter, also has an impact on the formation of spherical crystals. To prepare the spherical crystals, bridging liquid dichloromethane was added one time and dropwise to see the effect on crystallization.

Agitation speed

To prepare good-quality spherical crystals, the agitation speed during mixing plays an important role. The agitation speeds of 200, 400, 600, and 800 rpm were used to prepare the spherical crystals, and the outcomes were observed at different speeds.

Agitation time

Agitation time, which is a process parameter during spherical crystallization, should be optimized to ensure that good-quality spherical crystals are created. During the preparation of spherical crystals, 5-, 10-, 15-, and 20-minute time intervals were used.

To prepare the spherical crystals of the valsartan drug, a solution of valsartan and hydroxyl propyl cellulose was prepared in a good solvent. During the process, talc powder was added to prevent large lumps. This prepared solution was added to water, which is a bed solvent for the valsartan drug at a temperature of 5°C ± 1°C to start the process of crystallization (temperature difference concept). The bridging liquid was added, and mixing was carried out at different speeds to optimize the speed of mixing. This process was continued until the spherical crystals had formed. The prepared spherical crystals were filtered, dried at room temperature, and kept in the desiccator.

## Results

Flow property of valsartan

To check the flow pattern of pure valsartan drug, the angle of repose (using the fixed funnel method) was calculated. The result of the flow property of the drug is provided in Table 2.

**Table 2:** Angle of repose determination using the fixed funnel method

S. No.	Height of pile (h)	Diameter of pile (D)	2h/D	θ	Avg. θ
1	1.39	3.10	0.896	41.86	41.92º
2	1.40	3.12	0.897	41.89	
3	1.41	3.13	0.901	42.02	

The angle of repose of the spherical crystal is given in Table 3.

**Table 3:** Angle of repose of valsartan spherical crystals

Batch	Angle of repose
VST -1	31.40º
VST-2	27.23º
VST-3	35.73º

Selection of bridging liquid

The results for the use of a bridging liquid are given in Table 4.

**Table 4:** Bridging liquid and type of agglomeration

S. No.	Bridging liquid	Appearance
1	Toluene	No agglomeration
2	Chloroform	Clump formation
3	Dichloromethane	Spherical agglomerates

Mode of addition of bridging liquid

The results of the addition of a bridging liquid are shown in Table 5.

**Table 5:** Effect of mode of addition of bridging liquid

S. No.	Mode of addition	Observation
1.	Whole Amount	Shape of agglomerates irregular
2.	Drop-wise	Spherical agglomerates

Agitation speed

The results of the speed are given in Table 6.

**Table 6:** Effect of agitation speed

S. No.	Agitation speed (RPM)	Observation
1.	200	Large clumps
2.	400	Small agglomerates
3.	600	Spherical agglomerates
4.	800	Irregular-shaped agglomerates

Agitation time

The results of the agitation time are given in Table 7.

**Table 7:** Effect of the agitation time

S. no.	Agitation Time ( Min)	Observation
1.	5	Clump formation
2.	10	Incomplete formation of spherical agglomerates
3.	15	Spherical agglomerates
4.	20	Broken agglomerates

## Discussion

The angle of repose of pure valsartan drug, which is amorphous, was found to be 41.92º. This indicates the poor flow of the valsartan powder material. However, after the preparation of the spherical crystals for Batch VST-2, the angle of repose was less than 30º, which represents a good flowable powder. This type of crystalline powder can be used for direct compression in tablet preparation. Although the other two batches were better than pure drugs, they were still not good for direct compression due to the angle of repose being more than 30º.

The results for the choice of bridging liquid show that spherical crystals appear in dichloromethane, whereas in toluene, there was no agglomeration or chloroform clump formation. This indicates that dichloromethane is a good bridging liquid for the preparation of spherical crystals of valsartan.

The mode of addition of bridging liquid was studied. The bridging liquid dichloromethane was added during the process of spherical crystallization. It was added by one time (complete amount) and in another batch, it was added dropwise to see the effect. When all the bridging liquid was added at once, the shape of the spherical crystals was irregular, whereas spherically shaped crystals were formed when the bridging liquid was added dropwise.

At a low speed, the solvents and drugs were not mixed properly, which resulted in large clumps forming. However, at very high speeds, the crystals were broken down. Therefore, irregularly shaped spherical crystals were formed. At the speed of 600 rpm, which is the optimum speed, properly shaped crystals were formed.

When the mixing speed was optimized, clump formation took place after five minutes due to insufficient time for agglomeration. However, after 20 minutes, the crystals were broken down due to more attrition between the spherical crystals. At 15 minutes, which was the optimum time, properly shaped spherical crystals were formed.

The limitation of this study is that spherical crystals can be prepared only for amorphous drugs having an angle of repose of more than 40º. Another limitation is that this method is useful for solid dosage forms where crystalline properties are required.

The spherical crystals of valsartan were prepared using the crystallo-co-agglomeration technique because this technique allows obtained crystals to have a good spherical size which is good for direct compression. In this study, the different process parameters, such as the mode of agitation and agitation speed, were optimized in order to obtain a proper speed and mode so that this pilot plant scale-up process could be used in large-scale manufacturing processes.

## Conclusions

The pure drug valsartan was analyzed using the FTIR spectrometer and DSC analytical techniques, and these analytical methods confirmed the certificate of analysis of the drug. The spherical crystals of valsartan were prepared using the crystallo-co-agglomeration technique. N-dimethylformamide was used as a good solvent, whereas water was used as a bed solvent to prepare spherical crystals of valsartan. The good solution was added to the aqueous solution of hydroxypropyl cellulose in which talc was previously dispersed, and this talc powder is used to prevent clump formation. The flow properties of the pure drug and spherical crystals were checked using the angle of repose using the fixed funnel method. Out of three batches, the VST-2 batch was suitable as it flowed well. The prepared spherical crystals that flow well can be used to design an effective pharmaceutical tablet formulation, and the development cost of the formulation will be economical due to fewer manufacturing steps.
